# Nano- and Microsensors for In Vivo Real-Time Electrochemical Analysis: Present and Future Perspectives

**DOI:** 10.3390/nano12213736

**Published:** 2022-10-25

**Authors:** Alexander N. Vaneev, Roman V. Timoshenko, Petr V. Gorelkin, Natalia L. Klyachko, Yuri E. Korchev, Alexander S. Erofeev

**Affiliations:** 1 Research Laboratory of Biophysics, National University of Science and Technology “MISiS”, 119049 Moscow, Russia; 2 Chemistry Department, Lomonosov Moscow State University, 119991 Moscow, Russia; 3 Department of Medicine, Imperial College London, London W12 0NN, UK

**Keywords:** electrochemical sensors, analytical chemistry, bioanalytes, neurotransmitters, reactive oxygen species, nanoelectrodes, microelectrodes

## Abstract

Electrochemical nano- and microsensors have been a useful tool for measuring different analytes because of their small size, sensitivity, and favorable electrochemical properties. Using such sensors, it is possible to study physiological mechanisms at the cellular, tissue, and organ levels and determine the state of health and diseases. In this review, we highlight recent advances in the application of electrochemical sensors for measuring neurotransmitters, oxygen, ascorbate, drugs, pH values, and other analytes in vivo. The evolution of electrochemical sensors is discussed, with a particular focus on the development of significant fabrication schemes. Finally, we highlight the extensive applications of electrochemical sensors in medicine and biological science.

## 1. Introduction

In recent years, the growing need for more sensitive analytical and diagnostic tools for the identification of various analytes has led to significant advances in biomedical research. Current analytical methods based on carbon materials [1,2,3], nanoparticles [4], quantum dots [5,6,7], and fluorescent dyes [8] achieve high sensitivity with low invasiveness and allow real-time measurements within living systems.

Rapid developments and discoveries in healthcare in the near future, as a result of in vivo research, will open the possibility of a swift transition to personalized medicine. For example, the status of health in humans and other living organisms can be monitored through a number of parameters that are measured by implanted sensors. Such sensors would also allow study of the pharmacokinetics of drugs [9,10].

Electrochemical sensors are the most promising and effective techniques for real-time detection of analytes at a high spatial and temporal resolution [11]. Using such sensors, it is possible to study physiological mechanisms at the cellular, tissue, and organ levels and determine the state of health and diseases [12].

**Figure 1:**
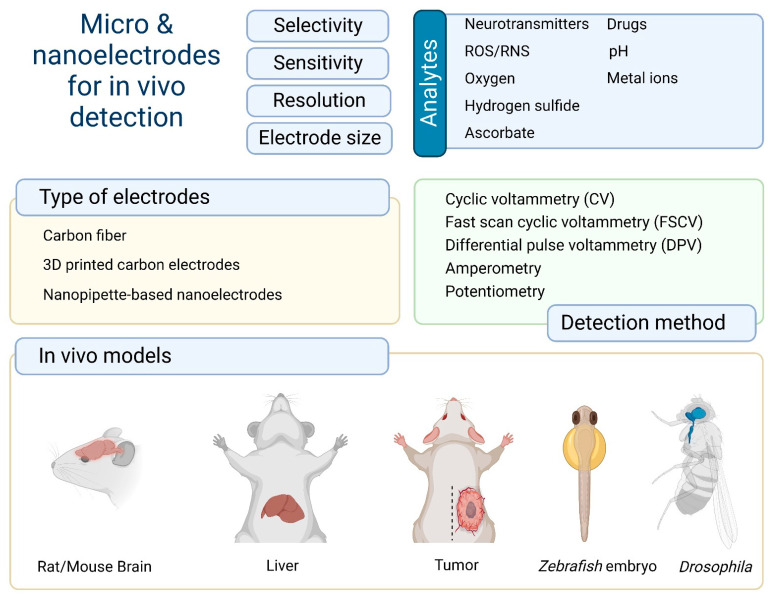
Brief summary of the types of analytes, electrodes, detection methods, and in vivo models for electrochemical detection.

Electrochemical methods have to satisfy a number of basic criteria for detection regarding sensitivity, selectivity, temporal resolution, and electrode size [13]. It is important to identify analytes at trace concentrations in biological systems and their dynamic concentrations. A high temporal resolution is critical and minimization of the electrode size allows analytes to be measured in living tissue with minimal damage. The most common bioanalytes are neurotransmitters [14,15], ascorbate [12], reactive oxygen and nitrogen species (ROS/RNS) [16], oxygen [17], and different metal ions [18] (Figure 1).

Today, there is a wealth of research that focuses on the in vivo detection of neurotransmitters [11,19,20,21,22,23,24]. The investigation of biological molecules involved in brain processes is an acute problem for the study of normal brain activity and to understand the pathophysiology of neurodegenerative and neuropsychiatric diseases. Abnormal levels of neurotransmitters usually indicate a violation of processes in the central nervous system (CNS). Therefore, in order to study these phenomena, highly sensitive, minimally invasive methods are needed to detect neurotransmitters that are capable of working in vivo and providing measurements in a specific area of the brain [25].

Fundamental research on neurotransmission toward understanding cellular signaling and communication between cells is of great interest. Microelectrodes can be extremely useful for studying the release of signaling molecules at the level of single cells, a process that requires extremely sensitive methodologies able to operate on very small scales [26,27].

When studying CNS, however, acute problems arise for electrochemical analysis: first, good stability of the electrodes during long-term measurements is required; second, sufficient selectivity is required in the determination of specific neurotransmitters; third, the electrode surface must be protected from protein adsorption; and fourth, sensitivity is important for detection at extremely low levels [28]. Such problems can be potentially avoided through the development of new approaches of electrode fabrication.

Another important area in biology is the study of oxidative stress. Oxidative stress has been implicated in aging [29], carcinogenesis [30], neurodegenerative disorders [31], and several other health-related conditions [32]. Although oxygen is an essential component of living organisms, biochemical reactions involving oxygen can lead to the formation of ROS/RNS, which can have a negative effect on the organism. There are many works devoted to the study of ROS/RNS at the cellular level [33,34,35,36,37,38,39]. In most cases, methods for the detection of individual ROS have been tested and successfully used in the detection of low levels in real time. It should be mentioned that ROS/RNS, as an apoptotic marker, can be involved in the mechanism of action of anticancer cytotoxic agents on tumor cells. Therefore, there is potential for such detection in the in vivo research of tumors and in determining the efficacy of anticancer drugs [40].

In vivo investigations of real-time drug pharmacokinetics are essential for tracking drug kinetics and evaluating drug efficacy, and their study using electrochemical techniques may accelerate the development of effective medical therapies. There are several recent reports on the in vivo monitoring of the antiepileptic drug lamotrigine [9], the anticancer reagent doxorubicin [9], and methylcobalamin [10] in the rat brain using electrochemical methods.

One promising approach for local measurements of some analytes in vivo is electrochemical techniques using nano- and microelectrodes based on carbon fibers (CFs) or glass nanopipettes. While sensors based on CFs have long been used, sensors based on glass nanopipettes are starting to be developed. New technologies for fabricating nano- and microelectrodes based on glass pipettes open great opportunities for the development of sensors. Nanopipettes offer several important advantages, including ease of manufacture, small size, and needle-like geometry. This makes them a suitable probe for scanning probe microscopy, including in scanning ion conduction microscopy (SICM) [41,42,43] and scanning electrochemical microscopy (SECM) [44,45].

In this review, we present recent advances within the last 4 years regarding the use of nano- and microelectrodes in various biomedical applications for in vivo real-time detection of different analytes. The evolution of electrochemical sensors is discussed, with a focus on the development of important fabrication schemes. Finally, we emphasize the extensive applications of electrochemical sensors in medicine and biological science.

## 2. In Vivo Applications of Nano- and Microelectrodes in Biological Systems

### 2.1. Neurotransmitters

**Figure 2:**
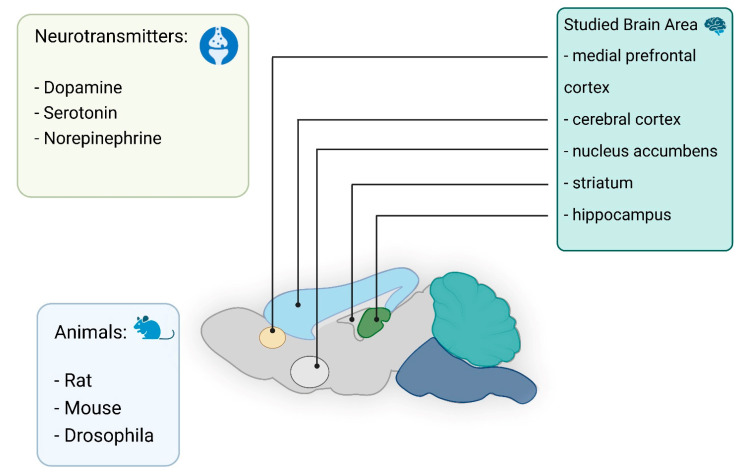
Summary of in vivo detection neurotransmitters in different area of the brain.

In vivo electrochemical investigations of neurotransmitters have long been conducted using microelectrodes. One of the first in vivo studies to measure catecholamines in brain tissue used a carbon paste electrode [46]. Nowadays, significant progress can be noted in the development of electrodes for neuromeasurements [11,22,23,24,47,48,49,50,51,52]. In particular, it is known that electrodes can be modified with graphene [53] to allow detection of dopamine (DA) without the influence of ascorbic acid (AA) and uric acid (UA). In vivo electrochemical analysis of neurotransmitters provides real-time insight into their functionality in the brain and body. This type of analysis can be very useful for monitoring, controlling chemical changes resulting from various diseases, and facilitating the diagnosis of diseases in the early stages [54,55]. To monitor the concentration of neurotransmitters in living systems, several different electrochemical methods are used, including amperometry, cyclic voltammetry (CV), and various pulse voltammetry methods. Neurotransmitters are detected by oxidation or reduction at the electrode surface. The analytes to be determined are limited by sensitivity, target selectivity, and device fouling and degradation over time. These include biogenic amines (DA, norepinephrine, and serotonin, etc.) and their metabolites in addition to AA and hydrogen sulfide. In this section, we describe the in vivo detection of neurotransmitters using nano- and microelectrodes (Figure 2).

Different measurement methods also affect the performance of the sensor in monitoring nerve signals. In general, microdialysis is easy to use and can be widely applied in long-term clinical neurochemical monitoring. However, it has a long delay and limited spatial resolution due to sampling characteristics [56]. In this review, we will consider the use of sensors for local measurement in vivo without the use of microdialysis systems.

In vivo monitoring of DA in the cerebral nervous system is a complex task, and resolving the associated issues is important to ensure reliable evaluation of the role of DA in physical and cognitive functionality. To reduce the invasiveness of the electrode, Ding et al. developed a nano-sized gold nanoelectrode for the detection of cerebral DA. A glass-sealed Au nanoelectrode was fabricated, with its tip modified using a cluster-like gold nanostructure and the working surface of the electrode modified using Nafion^®^. The developed electrochemical sensor was successfully used for amperometric monitoring of the DA level in the rat striatum; the concentration of released DA induced by nomifensine was estimated at 37 nM [57]. The modified sensor was specific and did not respond to UA, ascorbic acid AA, norepinephrine (NE), epinephrine, or 3,4-dihydroxyphenylacetic acid at physiological concentrations. The electrodeposited cluster-like gold nanostructure enhanced the current response. In this work, small-sized electrodes (<1 µm) were used, which made it possible to perform minimally invasive studies.

In order to improve the analytical performances of electrochemical sensors, a variety of electrode materials were combined with an even larger variety of nanomaterials during the years [58]. Electrode modifiers play an important role in separating spurious signals from DA signals. Therefore, electrode modifiers are currently being actively developed, including conductive polymer films [59,60] and graphene and its derivatives [61]. In particular, functionalized graphene containing various functional groups (e.g., carboxyl (–COOH), etc.) can significantly increase the sensitivity to the DA oxidation signal, mainly due to improved electrostatic interaction between the –COO^−^ group and DA^+^[62].

A typical working electrode for in vivo measurement is a carbon fiber microelectrode (CFME) made from a carbon fiber pulled in a glass capillary. CFMEs have been widely used for in vivo detection without modification due to their small size (5–7 µm diameter) and low RC constant, which results in rapid signal equilibration and ensures biocompatibility. Carbon is a suitable material for neurotransmitter measurements due to its surface structure having oxygen-containing functional groups and edge planes that allow the adsorption of dopamine and serotonin.

For example, endogenous DA release in the isolated adult *Drosophila* brain was measured using the fast scan cyclic voltammetry (FSCV) method involving CFMEs, and acetylcholine or nicotine was used as a stimulus for the release of endogenous DA. FSCV is a miscellaneous technology for in vivo neurotransmitter monitoring [21]. Detection of neurotransmitters in vivo can be quite challenging due to the low concentrations and transient events occurring on a subsecond time scale. Therefore, faster and more powerful methods are required for real-time neurotransmitter event detection. FSCV is a powerful electrochemical technique that provides a fast scan rate of ≥400 V/s, resulting in a high temporal resolution. The DA release stimulated by nicotine lasts much longer than the release stimulated by acetylcholine. CF has been used as working electrode with a diameter of 7 μm and was aspirated into a glass capillary [63]. Previously, these authors had fabricated carbon nanoelectrodes with tips ~250 nm in diameter. The electrochemical properties of such electrodes were first characterized using FSCV. Similarly to CF, carbon electrodes have a linear response for DA concentrations ranging from 0.1 to 10 µM and a limit of detection (LOD) of 25 ± 5 nM. Carbon nanoelectrodes are advantageous because they are an order of magnitude smaller in diameter than typical CFs and have a finely tuned geometry that facilitates penetration for localized measurements in specific areas of small organisms, such as the fruit fly brain [64]. However, all experiments to measure DA were performed ex vivo in mouse brain slices, although this technique can be used to detect DA in vivo [64].

**Figure 3:**
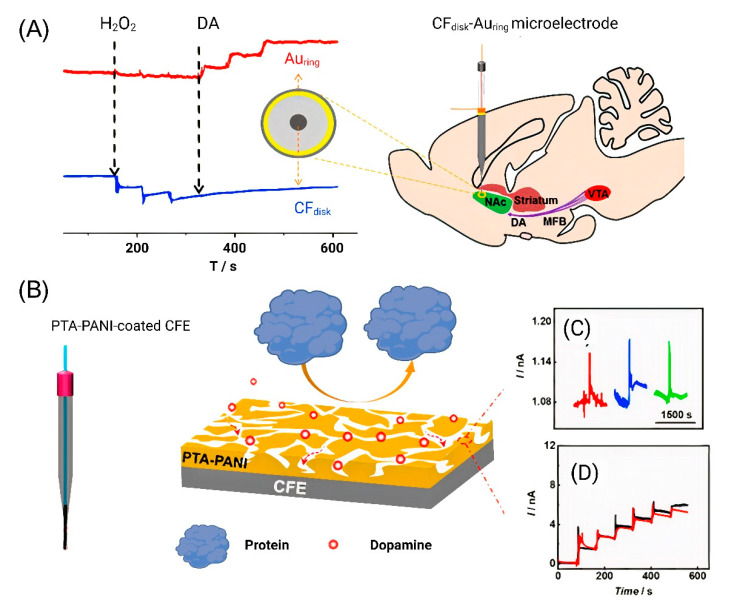
DA detection using modified CFE. (**A**) Simultaneous detection of DA and hydrogen peroxide in NAc. Reproduced from [65], copyright 2019 Elsevier. (**B**) Illustration of the structure of the PTA−PANI nanoporous membrane and the interface between PTA−PANI-coated CFE. (**C**) Amperometric response recorded with PTA−PANI-coated CFE implanted in the rat NAc by multiple stimulating MFB (each color corresponds to one independent stimulation). (**D**) Pre- (black curve) and post-calibration (red curve) curves obtained with PTA−PANI-coated CFE. Reproduced from [66], copyright 2019 American Chemical Society.

In addition to the determination of individual neurotransmitters, there is a trend toward the complex and simultaneous determination of several analytes. To determine DA, a ring-disk microelectrode was recently developed that made it possible to simultaneously determine DA and hydrogen peroxide. A microelectrode with a CF disk in the middle and nanometer gold ring was developed to simultaneously detect the two components. A Au electrode was used to detect DA [65]. The DA signal was obtained with electrical stimulation at 60 Hz ± 300 μA and 3 s in the nucleus accumbens (NAc) area. After the stimulation, a transient pulsed current change was recorded on the Au microelectrode (Figure 3A).

These same authors modified carbon-fiber electrodes (CFEs) to minimize biofouling-induced negative effects for in vivo analysis. Surface biofouling of the microelectrode has become a serious problem in in vivo experiments, as it significantly reduces the sensitivity of the sensor in the quantitative analysis. Polytannic acid (PTA)-doped nanoporous conductive polyaniline (PANI) membrane-coated CFE was developed, and PTA−PANI membranes have a hydrophilic electrode surface that prevents proteins from adhering. CFE was implanted in NAc of the rat brain, and the DA release was then monitored by bipolar stimulation the medial forebrain bundle (MFB) to validate the antifouling and detection ability of the PTA−PANI membrane. An increase in the current was observed after bipolar stimulation (Figure 3B). The PTA−PANI-coated nanoelectrode has high resistance to nonspecific adsorption of proteins in the cerebrospinal fluid and provides an accurate estimation of neurotransmitters in vivo [66].

Another trend in the development of modern methods for the determination of neurotransmitters has been their simultaneous detection. This task is extremely important, since a large number of neurotransmitters and metabolites are involved in neurotransmission. A method was recently developed that allows registration of changes in the oxygen concentration, DA, and electrophysiological activity in general in real time. The diameter of CFE was 5 μm, which allowed the authors to characterize the spreading depolarization (SD) in both superficial and deep areas of the brain in real time. Using FSCV, large changes in oxygen and DA release were observed in NAc. In this study, the authors were able to combine FSCV with electrophysiology to create a minimally invasive multimodal recording system capable of tracking SD waves. Maximum values of 8.2 ± 1.7 μM DA were obtained, which are in agreement with recordings made during SD in the striatum with less selective voltametric techniques and, more recently, with microdialysis [67].

For novel microsensors, it is difficult to simultaneously achieve a low LOD and high neural compatibility. A DA-sensitive fiber and potentiometric method was developed with an LOD of 5 nM. The sensitive fiber showed a wide linear range from 5 to 185 nM. The fiber was integrated for simultaneous monitoring of DA changes and electrical signals in the brain, with stable monitoring of in vivo DA changes for 8 weeks. CFE had no effect on the rate of excitation of neurons during the potentiometric test [68].

A new CFE surface functionalization strategy based on the assembly of aptameric cholesterol amphiphiles functionalized with an alkyl chain was recently presented. Noncovalent interactions of the cholesterol alkyl chain effectively immobilize aptamers on the CFE surface, which can be used to further determine DA in vivo [69].

The implantation of CFE into the brain tissue immediately results in nonspecific adsorption of proteins on the surface. This process can significantly affect the sensitivity and accuracy of the electrochemical method. To minimize electrode fouling, CFE was masked with leukocyte membranes (LMs). The reactivity of the electrode to target molecules was reduced to a small extent due to the membrane coating, but the loss of sensitivity of CFE with the LM mask was significantly reduced even after in vivo implantation for 8 h. The authors demonstrated an important approach when modifying the electrode surface with an antifouling LM-based material [70].

**Figure 4:**
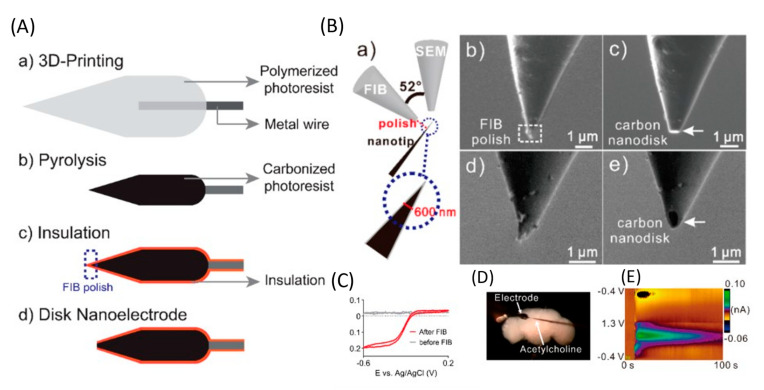
Application of 3D-printed electrodes. (**A**) Illustration outlining the fabrication of 3D-printed carbon nanoelectrodes. (**B**) Focused ion beam (FIB) polishing. (**a**) Illustration of the sample setup. The FIB detector is vertical to the nanotip sample, and the SEM detector has an angle of 52° to the FIB detector. FIB detector view (**b**) before and (**c**) after FIB polishing. SEM detector view (**d**) before and (**e**) after FIB polishing. (**C**) CV of the nanoelectrodes before and after FIB polishing in 10 mM Ru(NH_3_)_6_Cl_3_. Scan rate = 100 mV s^−1^. (**D**) Microscopy image of the adult brain with the 3D-printed nanoelectrode and a micropipette loaded with acetylcholine. (**E**) False color plot of DA detected after acetylcholine (2 pmol) stimulation. Reproduced from [72], copyright 2020 American Chemical Society.

The use of implantable microsensors in neuroscience research has undergone significant advancements. However, the geometry of most electrodes is limited by manufacturing methods, so new methods are needed for high-reproducibility mass production. Yang et al. developed a new method for fabricating an implantable microsensor using two-photon nanolithography followed by pyrolysis to fabricate microelectrodes with an electroactive carbon surface. The developed electrodes had an LOD for DA of 11 ± 1 (for spherical blanks) and 10 ± 2 nM (for cone-shaped blanks), high sensitivity to a variety of neurotransmitters, and high reproducibility and stability. Using spherical 3D-printed microelectrodes, DA was determined in a brain slice. This work is the first demonstration of 3D printing of carbon electrodes, and this method is promising for the mass production of individual, implantable neural sensors (Figure 4) [71].

Direct laser writing, a nano-3D printing approach, has enabled the manufacture of various sensors. However, submicrometer nanoelectrodes are required to detect neurotransmitters in tiny biological organisms or in synapses. Venton et al. used laser writing to 3D print free-standing carbon nanoelectrodes and use them as implantable sensors to detect neurotransmitters [72]. A separate standing nanotip was focused on a metal wire using a 3D printer and carbonized by pyrolysis. Carbon nanostructures were isolated by an atomic layer of an Al_2_O_3_ layer and polished to a disk shape using a focused ion beam. Carbon nanoelectrodes show promising electrochemical activity and detect DA signals in the brains of adult fruit flies. LOD for DA was 177 ± 21 nM, which is higher than LOD using CF (20 ± 4 nM) or a 3D-printed microelectrode (11 ± 1 nM) [71]. Nanoelectrodes were also used to detect DA in the mushroom body of adult *Drosophila* [71]. After stimulation at 10 s, 2 pmol of acetylcholine was applied, which resulted in an immediate rise in the current due to DA.

Understanding the chemistry of serotonin is limited by the complexity of in vivo chemical measurements. Serotonin is particularly difficult to electroanalytically detect in vivo due to its low extracellular concentrations. There is also a tendency of serotonin and its metabolites to affect the electrode surface [73].

Due to the presence of a large number of interfering analytes when examining in vivo signals, the signals from 5-HIAA, DA, and norepinephrine are filtered out [74]. In their next study, new ways of stimulating and measuring serotonin concentration were characterized in three brain areas in the CA2 region of the hippocampus, the medial prefrontal cortex, and the nigra pars reticulata, which is well known for being used in measuring serotonin via FSCV. It was shown that stimulation of the axons causes serotonin generation in the three measured areas of the brain [75].

In vivo measurements of serotonin in the CA2 region of the hippocampus of male and female mice showed that the serotonin concentration does not differ from the average value in female mice at different stages of the estrous cycle. In addition, mean male and female serotonin control signals and environmental levels were not significantly different. Serotonin measurements were performed using modified CFE [76].

**Figure 5:**
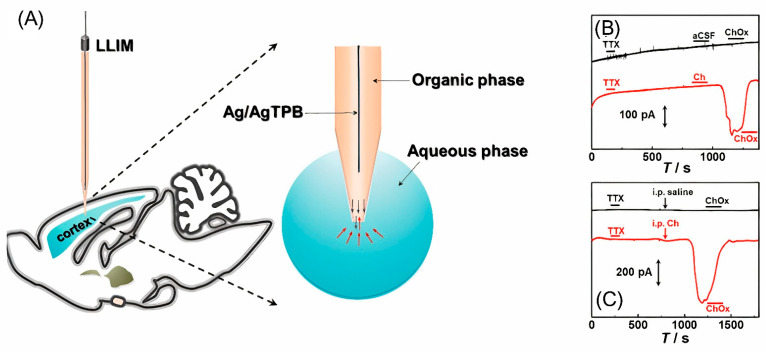
(**A**) Illustration of the liquid/liquid interface microsensor used in measurement of typical amperometric responses in the brain cortex of anesthetized rats during local microinfusion with (**B**) 100 ppm tetrodotoxin, pure aCSF and 1kU/L choline oxidase (ChOx) (black curve) and 100 ppm tetrodotoxin, aCSF containing 10 mM Ch and 1 kU/L ChOx (red curve), and (**C**) 100 ppm tetrodotoxin and intraperitoneal injection with 0.9% saline (black curve) or 334.8 mg/kg choline (red curve) and local microinfusion with 1 kU/L ChOx as labeled in the figure. Reproduced from [77], copyright 2021 American Chemical Society.

Recently, a liquid/liquid interface microsensor (LLIM) was developed to monitor redox inactive neurochemicals in the rat brain, i.e., choline (Figure 5) [77]. Based on differences in the solvation energies of choline in the cerebrospinal fluid (aqueous phase) and of 1,2-dichloroethane (organic phase), choline is recognized in the specific ion-transfer potential and distinctive ion-transfer current signals. LLIM demonstrated an excellent response to choline, with good linearity and selectivity and an LOD of 0.37 μM. In this study, a micropipette filled with an organic phase was used for the first time in the in vivo detection of choline. The use of a micropipette made it possible to improve the spatial resolution of the method.

Another analyte in the brain is hydrogen sulfide (H_2_S), which plays a key role in gas signal transmission, neuroprotection, and the regulation of physiological and pathological processes. Tian et al. demonstrated a novel potentiometric method for in vivo monitoring of hydrogen sulfide dynamics in the rat brain using AgNP-modified CF microelectrodes pretreated with Na_2_S (i.e., Ag_2_S/AgNP/CFE). Ag_2_S/AgNPs/CFE shows a good response potential to hydrogen sulfide in the range of 2.5–160 µM, with a detection limit of 0.8 µM. Due to the presence of Ag_2_S, Ag_2_S/AgNPs/CFE exhibits good selectivity for hydrogen sulfide, avoiding the influence of electroactive neurochemicals and analogs such as AA and cysteine in CNS. This good selectivity, combined with reversibility, protein fouling protection, and microelectrode biocompatibility, allows the detection of hydrogen sulfide in the rat brain during local Na_2_S microinfusion.

With the development of the field of machine learning, studies have appeared that use this approach to optimize electrode materials and detection conditions [78]. The use of machine learning approaches improves the sensor sensitivity, performance, and selectivity. For the first time, a deep-learning-based voltametric sensing platform was presented for the simultaneous recording of multiple neurochemicals (DA, AA) at the same location with a high spatiotemporal resolution in vivo. Using this algorithm, a single voltametric measurement can simultaneously detect unexpected relationships between neurotransmitters [79].

Hydrogen sulfide (H_2_S), a gaseous signaling molecule, has a regulatory function and is associated with various physiological and pathological processes. The biological activity and physiological functions associated with H_2_S may, however, occur or be mediated by hydrogen polysulfide. Hydrogen polysulfide can be formed during the oxidation of endogenous H_2_S in the presence of ROS and has a higher reactivity than H_2_S. The study of H_2_S_n_ is only at the initial stage, and many problems have not yet been resolved [80,81].

Recently, the authors developed an electrode with stability over long time periods based on CF modified using a gold and electrochemical probe with linear range of 0.25−20 μM for selective in vivo measurement of polysulfides. The electrochemical probe 4-(5-(1,2-dithiolan-3-yl)pentanamido)-1,2-phenylene-bis(2-fluoro-5-nitrobenzoate) was designed and built to determine H_2_S_n_. The biselectrophilic groups of the electrochemical probe can specifically recognize the two −SH groups in H_2_S_n_ and trigger the generation of an electroactive pyrocatechol moiety, resulting in a well-defined faradic current signal at ~0.24 V (vs. Ag/AgCl). It was shown that the H_2_S_n_ levels increased by 394.6 (from 1.09 ± 0.05 μM), 374.9 (from 1.05 ± 0.04 μM), and 275.5% (from 0.96 ± 0.02 μM) in the striatum, hippocampus, and cortex, respectively. This type of sensor is an example of a platform that has been successfully used to detect other electrochemically active molecules. In the future, the development and use of new electrochemical probes will make it possible to specifically determine the various types of biomolecules involved in physiological processes.

In conclusion, summary of the experimental studies related to neurotransmitters presented in Table 1. Over the past few years, the main problems that have been solved are related to preventing contamination of the electrode surface under in vivo conditions with an increase in sensitivity and temporal resolution. The main type of electrodes are CFs, which are an excellent platform for surface modification. However, the size of these electrodes is in the micrometer range, which affects the spatial resolution. The use of machine learning approaches has made it possible to take a new look at the processing of data obtained during the experiment for simultaneous determination of several types of neurotransmitters.

### 2.2. Ascorbate

Ascorbate plays an important role in various physiological processes. For example, it is believed that ascorbate serves as an antioxidant and neuromodulator of dopaminergic and glutamatergic neurotransmission and a cofactor for enzymes in various physiological and pathological processes [12,83]. AA has a direct impact on various biological processes such as collagen production, amino acid metabolism, wound healing, adrenal cortical hormone production, enzymatic amidation of neuropeptides, and scavenging of free radicals and singlet oxygen [84]. In neurons, the ascorbate concentration reaches 10 mM. The high intracellular concentration of ascorbate also indicates its indispensable role in various neuronal functions [83]. A decrease in the ascorbate concentration during ischemia or other damage makes cells vulnerable to oxidative damage [83].

In one recent work, CFEs were modified with single-walled carbon nanotubes (SWNTs) using electrophoretic deposition to measure ascorbate in vivo. Externally sheathing CFEs with SWNTs largely facilitates the oxidation of ascorbate, enabling selective measurements of ascorbate in CNS in vivo. A controllable and reproducible method was used to sheath CFEs with SWNTs with electrophoretic deposition. Using this approach, the CNT-sheathed CFEs showed improved electron-transfer kinetics toward ascorbate oxidation. The key feature of this method is in the measurement of basal ascorbate levels and the monitoring of ascorbate for 9 min after kainic acid infusion into the hippocampus to induce epilepsy [85].

These electrodes have been used to study ascorbate in the rat brain during SD propagation. Ascorbate release has been shown to be closely related to SD, with a significant increase in the ascorbate concentration in response to SD induction. This study highlights the potential of using the in vivo electrochemical system to understand the neurochemicals involved in many brain-related pathological processes [86].

In the study [87], the authors demonstrated a method for in vivo ratiometric detection of AA using CFE modified with carbonyl groups on graphene and PEDOT to avoid biofouling. The authors additionally removed epoxy groups to facilitate AA oxidation at −52 mV with increased sensitivity as confirmed by electrochemical investigation and density functional theory calculations. The results demonstrated a decrease in the AA levels to 217 ± 8 μM in the hippocampus and 220 ± 13 μM in the cortex (compared with control measurements of 257 ± 13 μM in the hippocampus and 256 ± 9 μM in the cortex on average). However, the AA levels in the striatum were statistically indistinguishable between Parkinson’s disease (PD) and normal mice, and the AA concentration was calculated to be 250 ± 16 mM. The integration of activated CFME in the deep-learning-based voltametric sensing platform has been demonstrated [79] for application in multi-analyte detection (representing a classical neurotransmitter and including AA detection).

Thus, authors have tried to address four main problems in the detection of AA: (1) High overvoltage of AA, which significantly complicates the selective monitoring of AA; (2) the change in the AA levels in some physiological processes is small enough such that high sensitivity is required; (3) the chemical complexity of the brain requires that electrochemical methods have good stability and reproducibility; (4) and the adsorption of biomolecules on implanted microelectrodes requires in vivo electrodes to have an excellent antifouling ability.

### 2.3. ROS/RNS

ROS and RNS are known metabolic products. The detection of ROS/RNS in biological models has recently been reviewed [14,88]. In relatively low concentrations, they play a multifaceted role in the regulation of a number of physiological processes. Overproduction of ROS/RNS contributes to the pathogenesis of a variety of physiological disorders, including but not limited to cardiovascular disease, neurodegenerative disease, and cancer.

The task of detecting and quantifying ROS and RNS is replete with challenges related to the inherent characteristics of these species: short lifespan, low physiological concentration, and high reactivity [89]; thus, sensitivity and selectivity are essential. Electrochemical detection and monitoring of ROS/RNS is a rapidly evolving field fueled by numerous research publications.

Recently, Dumitrescu et al. measured, for the first time, the distribution of NO in the gut of live zebrafish (*Danio rerio*) embryos treated with resveratrol and rosuvastatin. NO measurements were performed with CFEs, which allowed quantitative monitoring of various segments of the intestine in real time. In the presence of resveratrol and rosuvastatin, the intestinal NO concentration decreased by 87%. These results indicate the presence of different micromolar concentrations of NO along the intestinal tract of zebrafish embryos. The usefulness of CF microelectrodes for the quantitative measurement of NO release at the single-organ level in individual zebrafish embryos has also been demonstrated The key feature is modification of CFs with a selected NO-specific catalytic material, nickel (II) phthalocyanine-tetrasulfonic acid tetrasodium salt [90].

A DNA scaffold for fabricating a transducer with precise and engineerable structures was recently developed. In particular, G-rich sequences can form a special DNA structure called the G-quadruplex and then coordinate with porphyrin-like biocatalysts to form a G4-porphyrin. The assembled DNA-G4/porphyrin hybrid represents a favorable electrocatalytic transducer for NO. The fabricated microsensor has a high sensitivity (LOD, 13.5 pM), a wide detection range (100 pM–5 µM), and good selectivity. Thus, the release of NO by cells and tissues can be directly and accurately monitored. It was shown that the nature of NO released by various lines of cancer cells could be determined in learning about its dynamics in the in vivo tumor microenvironment [91].

NO was detected in the living rat brain using carbon fiber microelectrodes covered with nickel porphyrin and this fluorinated xerogel. These microsensors were insensitive to interfering redox molecules, and their performance surpassed that of similar microelectrodes coated with a Nafion screening layer. In vivo, these electrodes could detect brain NO released in the rat parietal cortex in response to local microinjection of the glutamatergic agonist N-methyl-d-aspartate (NMDA). The NMDA-evoked NO release peaked at 1.1 μM and lasted more than 20 min [92].

The electrochemical hydrogen peroxide reduction reaction has been recognized as an effective approach for detecting hydrogen peroxide. Using Cu_1_/C_3_N_4_ as the single-atom Cu electrocatalyst, microsensors were developed with a good response specifically to hydrogen peroxide and not to O_2_ or other electroactive neurochemicals. When implanted in the brain of a live rat, the microsensor exhibited excellent in vivo sensing characteristics, making it suitable for real-time quantification of hydrogen peroxide dynamics [93].

A selective and accurate electrochemical biosensor with a high spatial and temporal resolution was developed for real-time monitoring of ONOO^−^ levels in different regions of the rat brain following global cerebral ischemia. First, a new organic molecule, 4-(S-(6-mercaptohexyl)benzothioate-6-yl)-7-(diethylamino)-2-(4-(piperazinyl diferroformamide-1-yl)phenyl)chromenylium (HEMF) with a specific recognition group toward ONOO^−^ and an electroactive group (ferrocene) was designed and synthesized for the determination of ONOO^−^ with high selectivity, and it demonstrated good linearity for concentrations of ONOO^−^ in the range from 20.0 nM to 2.0 μM, with an LOD as low as 12.1 ± 0.8 nM. Finally, combined with the unique properties of CFE, including a high spatial resolution (10 μm) and good biocompatibility, the developed ratiometric electrochemical biosensor with a high temporal resolution was successfully applied in the detection of ONOO^−^ in three regions of the rat brain following cerebral ischemia [94].

Fouling of the electrode surface is a key problem that limits the practical application of sensors under challenging conditions in vitro and in vivo. Surface contamination greatly affects the sensitivity, stability, service life, and reproducibility of electrochemical sensors. However, this problem is currently receiving great attention throughout the world. Recently, an electrochemical H_2_O_2_ sensor with a nanoporous silica membrane as an antifouling layer and platinum nanostructures inside the nanopores was developed as a catalyst to improve its sensitivity. The antifouling properties were demonstrated by implanting the electrode into the rat brain, where it successfully functioned for 1.5 h [91].

A CF sensor modified with 1,3-phenylenediamine has been developed [95]. Electrodeposition of this polymer allows the creation of a membrane that rejects prominent molecular interferences that can be misidentified as H_2_O_2_. This membrane is widely used as a coating in both microdialysis samples [96] and electrochemical sensors [97]. Using an unmodified, uncoated microelectrode, a rapid increase in the DA concentration as a result of vesicular ejection was detected in addition to a small signal, which was presumably attributed to H_2_O_2_. A slight increase in H_2_O_2_ was recorded in response to stimulation using the modified sensor, but no DA signal was observed (because it was effectively eliminated by the membrane).

The monitoring of ROS/RNS levels with high sensitivity in tissues is also essential for the early stage diagnosis of cancer diseases. Vaneev et al. showed that a new method developed in our lab can measure the ROS response to chemotherapy in tumor-bearing mice in real time. ROS levels were measured in vivo using a platinized carbon nanoelectrode inside the tumor at different depths in response to doxorubicin. This work provides an effective new approach for the measurement of intracellular ROS using platinized nanoelectrodes [40].

In conclusion, summary of the experimental studies related to ROS/RNS presented in Table 2. Despite important advances in the field of sensors, some challenges related to ROS/RNS sensitivity still need to be overcome before commercialization of the related commercialize electrochemical technologies and their application in clinical practice. For clinical use, factors such as safety, availability, high throughput, rapid analysis, and reproducibility must be seriously considered. The manufacturing process should be improved to be safe and controlled such that industrial production is allowed and sensor-to-sensor reproducibility is ensured. In addition, sensors can be improved to increase the speed and accuracy of analyte detection and prolong their life, and the accompanying communication systems must also be improved to ensure the security of user data.

### 2.4. pH

Monitoring dynamic changes in pH is an acute challenge for understanding physiological processes. Acid–base disorders are closely linked with the occurrence and progression of diseases such as cystic fibrosis [98], ischemia [98,99], psychiatric disorders [100], diseases of organs of the gastrointestinal tract [101], and cancer. The tumor microenvironment is most susceptible to pH changes [102]. The pH in tumor cells and the tumor microenvironment play an important role in the development and treatment of cancer. It has generally been accepted that both extracellular and intracellular pH values in tumors are acidic and lower than in normal cells. However, it was recently shown that the intracellular pH (pH_i_) of cancer cells is neutral or even slightly alkaline compared with normal tissue cells.

There are currently several methods for measuring pH_i_ and pH_e_ in cells, including pH-sensitive nuclear magnetic resonance spectroscopy [103], positron emission tomography [104], radiotracers, magnetic resonance imaging [105], optical imaging [106], and different types of electrochemical sensors.

Recently, potentiometric sensors based on MoS_2_ and PAN have been developed that allow monitoring of the dynamics of pH changes in vivo with high reliability and selectivity [107]. The polyaniline in the sensor is used as an active pH-sensitive material. MoS_2_ has been used due to its large surface area, good biocompatibility, and excellent electrochemical properties. The pH microneedle was prepared through layer-to-layer assembly of MoS_2_ and PAN. To adjust the pH environment in vivo, rat brains were injected with NaH_2_PO_4_ and Na_2_CO_3_, respectively, evoking pH changes in the cerebrospinal fluid.

A similar type of electrochemical sensor based on poly(melamine) films for the ratiometric monitoring of pH in subacute PD mouse brains was developed. Poly(melamine) films were prepared from a simple electropolymerization approach in a melamine-containing solution, serving as the selective pH recognition membrane undergoing a 2H^+^/2e^−^ process. Meanwhile, electrochemically oxidized graphene oxide (EOGO) produced a built-in correction signal that helped avoid the environmental interference of the complicated brain systems. The potential difference between the peaks generated from EOGO and poly(melamine) gradually decreased with the increase in aqueous pH from 4.0 to 9.0, constituting the foundation for detection by the ratiometric electrochemical microsensor [108].

Recently, a micrometer sensor has been developed that allows determination of the pH inside a rat brain with a good time and spatial resolution [109]. Polyimidazole-based polymers were first attached to the inner wall of micropipettes via a radical polymerization reaction. The association/dissociation of imidazole protons results in a pH change that will increase/decrease the surface charge density, which consequently results in a change in the ion current for pH analysis.

The sensor showed good linearity and reversibility in the pH range from 5.8 to 8.0, with an average response time of ~ms. A sensor was implanted in the cerebral cortex to monitor changes in pH during disturbance of the acid–base balance by breathing pure CO_2_ gas. Breathing pure CO_2_ gas rapidly reduced the pH by approximately 0.10 ± 0.05.

The authors made a great contribution to the development of a pH sensor, but at the moment, it is impossible to accept the low invasiveness of the measurement process, especially when the scientific goal requires a deep brain measure, such as pH measurements in the hypothalamus/striatum/cortex.

### 2.5. Oxygen

The main function of oxygen is its participation as an oxidizer in redox reactions in an organism. It is difficult to overestimate the importance of brain processes that depend on correct oxygen saturation of the brain. Therefore, most recent research has mainly aimed to develop novel/improved electrochemical sensors for in vivo measurements in the brain or to study the deep relationships between oxygen and various processes using existing electrodes.

The first study investigated the properties of a “naked” CFME electrode functionalized with a silica nanoporous membrane, which could effectively protect the surface from biofouling and, meanwhile, retain permeability to O_2_ [110]. The synthesized membrane was grafted using electro-assisted self-assembly. The authors studied the structure and morphology of the deposited membrane and demonstrated its permeability to oxygen. The applied membrane had an improved signal-to-noise ratio and increased electrode stability for long application periods. The antibiofouling capacity (expressed as the relative current stability in the conferred bovine serum albumin) of the modified electrode was 85% of the baseline value after 60 min vs. 55% of the baseline value for unmodified CFME after 30 min.

**Figure 6:**
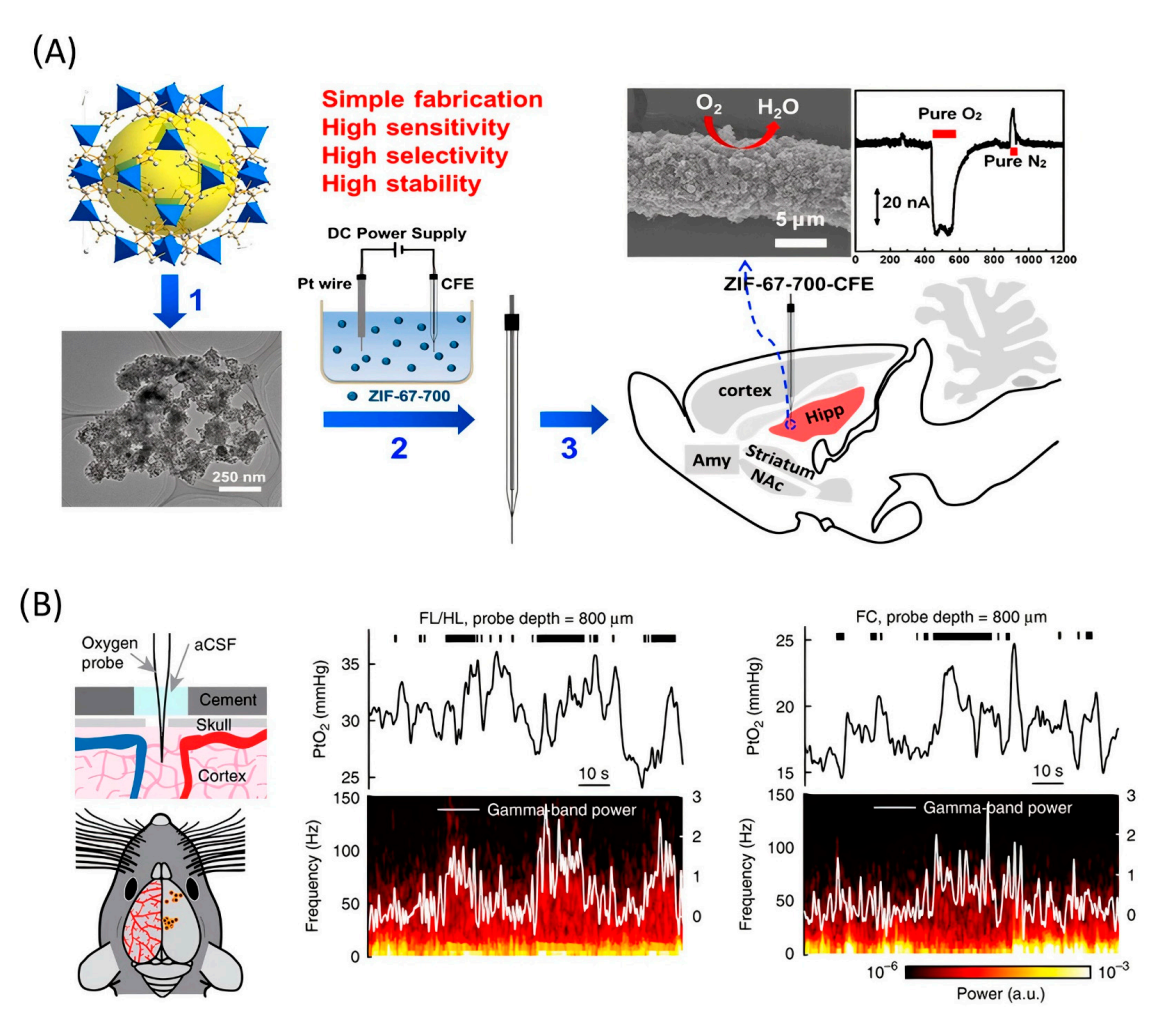
(**A**) Application of Co/N/C catalysts for in vivo monitoring of oxygen. (1) Co/N/C catalysts were obtained by pyrolyzing ZIF-67 at 700 °C. (2) Sensor fabrication by electrophoretic deposition of catalyst onto a carbon fiber microelectrode. (3) As-fabricated sensor applied for in vivo monitoring of O_2_. Reproduced from [111], copyright 2019 American Chemical Society. (**B**) Left, experimental setup and measurement sites. Center, example traces showing the PtO_2_ responses to locomotion at sites 800 µm below the brain surface in the forelimb/hindlimb representation of the somatosensory cortex (FL/HL) and frontal cortex (FC). Top, black ticks denote binarized locomotion events. Middle, PtO_2_ responses to locomotion. Bottom, example of data showing a spectrogram of LFP (local-field potential). Reproduced from [112].

The subsequent study demonstrated the application of functionalized CFME by metal/nitrogen/carbon (M/N/C (M = Co or Fe) nanocomposites for the in vivo electrochemical monitoring of oxygen [111]. The main reason for using non-Pt-based functionalization material is the higher stability and resistance to surface poisoning in complicated physiological environments. The author used M/N/C nanocomposites that had been prepared by pyrolysis of the zeolitic imidazolate framework. CFME surface modification was carried out by electrophoretic deposition (Figure 6A). Measurement stability was confirmed in the presence of several electrochemically active chemical species in the cerebral system and surface contamination proteins. The electrode sensitivity was 0.0012 μA*μM^−1^. This work concluded with in vivo measurements, which show a basal level of O_2_ in the rat hippocampus of 30 ± 10 μM (n = 3).

A Clark-type electrode was used to measure cerebral oxygenation in [112]. A notable feature of the work is that the authors used awake, head-fixed mice as the object of measurement (Figure 6B). The oxygen content in the cerebral cortex of mice during locomotion was measured using polarography, spectroscopy, and two-photon phosphorescence lifetime measurements of oxygen sensors. The authors found that locomotion significantly and globally increased cerebral oxygenation specifically in areas involved in locomotion and in the frontal cortex and the olfactory bulb. The increase in oxygenation persisted when neural activity and functional hyperemia were blocked, occurred both in tissue and in arteries that feed the brain, and was closely correlated with the respiration rate and the phase of the respiration cycle.

Despite the small number of studies on in vivo oxygen determination over the past four years, some notable advances have been achieved. Extensive experiments have been carried out to determine the influence of external factors on the developed sensors mostly before application for the collection of real in vivo measurements. During in vivo measurements, electrodes are often subjected to severe external influences that change their characteristics. For example, this is clearly seen in a work showing O2 calibration curves obtained before and after in vivo implantation in the rat brain for 2 h [110].

### 2.6. Metal Ions

Another important task is the detection of metal ions in vivo. This is primarily due to metal contamination of the environment and, as a result, more frequent cases of metal imbalance in living organisms [113,114]. The imbalance of metals in a living organism can be congenital or associated with hereditary diseases such as Wilson, Menkes, and Alzheimer’s diseases [115]. Secondly, the development of novel drugs containing various metals requires an assessment of their penetrating/accumulative ability [116,117]. All these aspects emphasize the need for increased attention in resolving the issue of quantitative control of metal in vivo.

Ratiometric measurement is a key technique in the in vivo detection of metals. Ratiometric electrochemical sensors record a double electrical signal from the measurement area, and the quantitative determination is based on the ratio of these two signals, which significantly improves the measurement accuracy [118].

Over the past few years, several articles have considered ratiometric sensors for use in in vivo measurement [119,120,121,122]. The first study demonstrated changes in pH and compared the quantity of copper ions in normal and Alzheimer’s disease (AD) rats [119]. The researchers used a glassy carbon (GC) as a platform for electrode fabrication, which was functionalized by a series of *N*,*N*-bis(2-[2-(ethylthio)ethyl])-based (NS4s) derivatives for the specific recognition of Cu^+^. At the same time, 9,10-anthraquinone was used in selective modification of a pH sensor using 2,2′-azino-bis(3-ethylbenzthiazoline-6-sulfonic acid) modifier as an internal standard.

This system was used to determine the concentration of copper ions as 2.35 ± 0.22 μM in the striatum, 2.08 ± 0.20 μM in the hippocampus, and 1.75 ± 0.13 μM in the cerebral cortex. Cu^+^ levels increased by ~1.8 times in the AD mouse model. These values closely match the data of another study [123]. However, it was reported that real-time measurements and reuse of sensors were not possible. In vivo copper ion sensors were further developed in the research of Gu et al. (2019), where a ratiometric sensor was fabricated for the detection of Cu^2+^ in real time with the possibility of reusing electrodes [120]. The working electrode was prepared by functionalizing GC with branched polyethyleneimine to bind Cu^2+^, and the auxiliary electrode was modified by 6-(ferrocenyl) hexanethiol, which served as the inner reference moiety in the regeneration process. Gu et al. (2019) then measured the concentration of intrinsic Cu^2+^ in the rat brain before (~1.7 μM) and after (~5 μM) global cerebral ischemia.

Other metal ions are also involved in the processes of neurodegenerative diseases. Zhang et al. presented a ratiometric electrochemical sensor based on a Au−C≡C bond surface for real-time mapping and accurate quantification of Fe^2+^ in the brains of a live AD mouse [122]. The author used a carbon CF microelectrode as a base followed by gold precipitation on the electrode surface. Furthermore, the detection electrode surface was functionalized using 2,5-furandicarboxylic acid (FDCA) molecules, which specifically react with Fe^2+^. The functionalization of the gold surface was carried out in three ways, and as a result, it was determined that Au–C≡C has improved stability and electrochemical characteristics.

This system was used to determine the Fe^2+^ concentration in the hippocampus (1.18 ± 0.13 μM), cerebral cortex (1.09 ± 0.09 μM), and striatum (1.30 ± 0.15 μM) of the brain of control mice under normal conditions. It was noted that the concentration of Fe^2+^ in three brain parts of the AD mice was increased by an average of 108.6 ± 4.8%. In addition to this, the authors studied the relationship between Fe^2+^ and Ca^2+^ channels. Thus, this research demonstrates a strategy for developing stable and sensitive sensors based on Au–C≡C and establishes a pathway for Fe^2+^ uptake by neurons.

Another nonratiometric sensor was presented by Zhao et al., who showed a sensor system for the simultaneous determination of glutamate and calcium ion in the rat brain [124]. The dual function microelectrode consisted of a Pt microelectrode, which was functionalized by the glucose oxidase enzyme NP for glutamate detection, and CFME, which was fabricated by immersing CFME in a Ca^2+^-selective cocktail (ETH1001, PVC, o-NPOE, THF). Changes in the glutamate and calcium concentrations in rats were determined during SD (5.0–7.1 μM for Glu and 620–730 μM for Ca^2+^) and ischemia (19.2–26.6 μM for Glu and 840–960 μM for Ca^2+^). The authors demonstrated a relatively close connection between Ca^2+^ and glutamate during SD and the ischemic processes using a developed sensory system.

CFME modified with gold particles was electrodeposited and alternately wrapped with graphene oxide microbands in which the exposed gold particles provided active sites for the modification of recognition molecules [125].

The researchers used graphene oxide as an antibiofouling coating. To specifically recognize Ca^2+^ in the brain, three kinds of organic molecules with two main parts were used: recognition groups for selective determination of Ca^2+^ and terminal groups for the molecules self-assembled onto the electrodes. Based on biofouling, reversibility, and specificity tests, the authors developed a sensor with a linear range from 10.0 µM to 31.6 mM while LOD was estimated at 5.91 ± 0.33 µM. A stable electrode can be integrated into a microchip to simultaneously monitor the concentration of Ca^2+^ at different depths in various brain regions within the same mouse.

The detection of metal-containing complexes is also an important task. In the research of [18], the authors used an easily prepared platinized carbon nanopipette to detect platinum-based antitumor compounds using scanning ion-conductance microscopy with a confocal module. Prior to the in vivo experiments, the authors demonstrated quantitative direct detection of Pt(II) species in breast adenocarcinoma MCF-7 cells and 3D tumor spheroids treated with cisplatin and a cisplatin-based prodrug. Increased accumulation of cisplatin-based prodrug compared with cisplatin was confirmed through the in vitro measurement of both. This study concluded with in vivo measurement in a mouse model of mammary carcinoma EMT6. The prodrug was found to penetrate more deeply than cisplatin. This research demonstrates a minimally invasive, real-time electrochemical technique for the study of platinum-based drugs that enables the possibility of determining the prospects of platinum-containing drugs by assessing their penetration using in vitro and in vivo models [126,127].

The development of new and the improvement of existing electrochemical methods for quantitative in vivo detection of metal ions is a big step toward understanding intratumoral/intracerebral processes. The data obtained with these sensors allow us to explore the study of cancer progression, Alzheimer’s, Wilson, and Menkes diseases in more depth and the processes of global ischemia, which are often a consequence of neurodegenerative diseases. We expect that future electrochemical sensors for in vivo applications will become less invasive (by decreasing the size of the electrodes from micrometer to nanometer) with ubiquitous online detection and simultaneous detection of multiple ions.

### 2.7. Other Analytes

In this section, we include articles aimed at the development of electrochemical sensors that did not fall into the main sections but, at the same time, contribute considerably to studies of the functioning of living organisms.

Over the past 4 years, special attention has been paid to the development of a stable electrode for the in vivo measurement of chloride ions regarding their association with channelopathies diseases and the regulation of numerous physiological functions and cellular parameters such as membrane excitability, cell volume, charge balance, and resting potential [128,129]. A research team led by Hui Gu presented two articles aimed at in vivo ratiometric detection of chloride ions using modified solid state electrodes based on CF [130,131]. The main idea of the two investigations was the modification of CFME by Ag. Ag-coated sensors were used for the selective determination of Cl^−^, which was attributed to the characteristic electrical chemical reaction between Ag and Cl^−^. Chloride ions were detected in the range of 1 to 700 [130] and 1 to 300 mM [131]. As a result, the C57BL mouse brain was estimated (84.2 [130] and 95.5 mM [131]). The results of sensor application demonstrated that the Cl^−^ levels were increased in the cortex, whereas they were decreased in the hippocampus of the model of the PD mouse brain [130]. Thus, these studies demonstrate a simple solution for designing Ag-coated electrodes (avoiding the complicated processes of metal reduction) suitable for in vivo monitoring of chloride ion levels.

Medicines play a key role in the treatment of patients with various diseases. After its administration, a drug is distributed to various systems in the body. It is worth noting that the simultaneous determination of the pharmacokinetics of the drug and its pharmacological effects in the in vivo microenvironment in real time is important for evaluating the effectiveness of drugs. Electrochemical methods make it possible to track the behavior of chemical compounds over time with a high time resolution [132]. There are several recent studies in which attempts have been made to directly identify drugs within the body. In particular, the methylcobalmin content inside two different parts of a live guinea pig was determined in real time. Boron-doped diamond electrodes were used to measure the levels inside the cochlea and inside the leg muscles [10]. It is worth noting that this direction of research awaits further development. The main work on the electrochemical determination of drugs in vivo has been performed using boron-doped diamond electrodes.

## 3. Antifouling Coatings

Probe biofouling is a problem that must be overcome when developing in vivo detection technology. Unwanted molecules tend to attach to the surface of the platform through several types of interactions, such as hydrophobic, electrostatic, and intermolecular forces, resulting in a dense layer that forms passively on the electrode and blocks the recognition elements and the electrode itself. The fouling layer not only prevents physical contact of the analyte with the electrode but also directly prevents electron transfer through electrochemical reactions [133,134].

**Figure 7:**
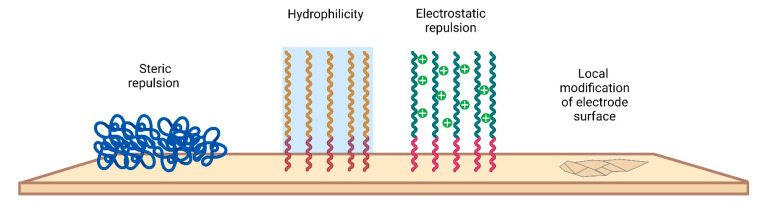
Different antifouling approaches for electrode surfaces.

Various approaches have been taken to minimize biofouling of the electrode surface. A current strategy to address fouling issues is to integrate antifouling materials on the electrode surface to effectively minimize nonspecific attachment. The main requirements that materials must meet concern biocompatibility, inertness, and stability. By interacting with the electrode surface, antifouling agents include a wide range of materials, most of which are aimed at increasing the antifouling resistance of electrodes [135,136]. There are several modification strategies that involve either changing the chemical properties or creating a physical structure (Figure 7).

In general, antifouling materials resist biofilm formation by ensuring that the interaction of the surface with the biological environment is reversible. Since antifouling materials have a low interface energy with water, contaminants are not deposited on the electrode surface. In addition, for high-molecular-weight PEGs, steric repulsion can be used to explain the mechanism of protein stability. PEG is the most widely used antifouling interface design material and is considered the “gold standard” of antifouling performance [137]. To create an antifouling surface, it is necessary to modify the surface so that it allows water molecules to bind to the surface over other materials. Antifouling surfaces must be very hydrophilic. The induced hydrophilicity is able to reduce nonpolar interactions between functional groups and these surfaces. Thus, the electrical neutrality of antifouling materials can help reduce electrostatic interactions with charged contaminants [138]. In addition, antifouling materials can prevent adsorption by steric hindrance. In this section, we only briefly mention some examples of the use of electrode modification materials used in vivo in recent times.

Feng et al. developed a nanoporous membrane of polymerized tannic acids and polyaniline as a new antifouling coating for electrodes used in in vivo electrochemical experiments [66]. Polyaniline is widely used in biosensing because of its controlled conductivity, good environmental stability, and excellent redox activity, which enable fast mass transfer through the nanochannel and fast electron transfer and/or exchange along the conducting framework [138]. Nanoporous conductive polymers will potentially reduce nonspecific protein adsorption. To obtain a nanoporous and conductive structure, polyaniline was subjected to electropolymerization, and PTA containing several phenolic hydroxyl groups was also used. PTA has been used as a dopant to improve the electrochemical activity of PANI at physiological pH because PANI loses its electrochemical activity above pH 4.0. The prepared PTA–PANI membrane forms a hydrophilic electrode surface and prevents protein penetration to the electrode surface.

A novel sensing interface was developed through robust polyDA-engineered biointerfacing to tailing zwitterionic molecules via Michael addition, which can resist nonspecific protein binding in complex biological fluids while the interfacial electron transfer and electrochemical stability of the electrode are enhanced. In addition, this sensing interface can be integrated with a tissue-implantable electrode for in vivo analysis with an improved sensing performance, preserving ca. 92.0% of the initial sensitivity after 2 h of implantation in brain tissue, showing low acute neuroinflammatory responses and good stability in both normal and PD rat brain tissue [139].

**Figure 8:**
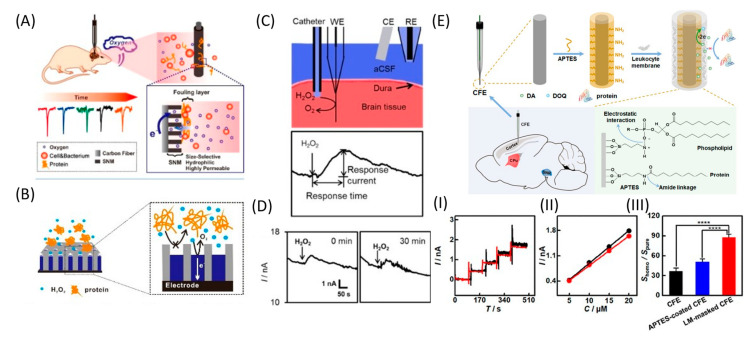
Different antifouling approaches. (**A**) Illustration of hydrophilic, highly permeable, and antibiofouling SNM-coated CFME for continuous monitoring of O_2_ in the rat brain. Reproduced from [110], copyright 2019 American Chemical Society. (**B**) Schematic diagram of the low-fouling Pt@SNM-coated electrode for H_2_O_2_ detection. (**C**) Scheme of intermittent in vivo monitoring of H_2_O_2_ by Pt_80_@SNM/CFME in the rat brain. (**D**) In vivo monitoring of H_2_O_2_ by Pt_80_@SNM/CFME implanted in the cortex of the rat brain for 1.5 h, whereby 1 μL of 0.5 mM H_2_O_2_ was injected for 20 s every 30 min. Reproduced from [91], copyright 2020 Wiley-VCH Verlag GmbH & Co. KGaA. (**E**) Scheme of CFE modification with leukocyte membrane for in vivo detection DA. (**I**) Typical amperometric responses with the LM-masked CFE toward successive additions of DA (each addition was 5 μM) in pure aCSF (black curve) and homogenate-containing aCSF (red curve). (**II**) Calibration curves obtained with the LM-masked CFE in pure aCSF (black curve) and homogenate-containing aCSF (red curve). (**III**) Ratios of the sensitivity in homogenate-containing aCSF to those in pure aCSF; n = 4. The asterisks (****) indicate significant differences, *p* < 0.0001, one-way ANOVA. Reproduced from [70], copyright 2019 American Chemical Society.

Another electrode coating is a nanoporous silicon dioxide membrane (SNM) consisting of uniform, densely packed, and vertically aligned nanochannels on the CFME surface (Figure 8A,B). The membrane effectively protects the surface from biological fouling and, at the same time, maintains O_2_ permeability. Compared with pure CFME, the implanted modified electrode could continuously monitor O_2_ under in vivo conditions, showing excellent current stability and a fast response for up to 2 h. Moreover, given the high permeability, selectivity, and biocompatibility of SNM, this membrane can be used for long-term in vivo monitoring of O_2_ and other neurotransmitters [110]. The same authors further developed the SNM antifouling membrane to fabricate low-fouling H_2_O_2_ sensors for the electrochemical detection of H_2_O_2_ in complex samples. Pt nanostructures were deposited into the pores of the membrane to improve the sensitivity and selectivity with respect to H_2_O_2_. Such Pt@SNM/CFME functioned stably in the rat brain for up to 90 min, with successful detection of the H_2_O_2_ content in the brain cortex (Figure 8C,D) [91].

One effective strategy for minimizing electrode biofouling is electrode masking using LM. The LM masking endows CFE with a highly hydrophilic surface that becomes highly resistant to nonspecific protein adsorption. The reactivity of the electrode towards target molecules is reduced to a small extent due to the membrane coating, but the loss of sensitivity of LM-masked CFEs is significantly reduced even after in vivo implantation for 8 h (Figure 8E) [70].

Erythrocyte membrane was deposited on Ag/AgCl/IL electrodes to significantly improve the biocompatibility and potential stability of the electrodes [140]. The erythrocyte membrane was extracted from rat blood and applied to chemically modified Ag/AgCl electrodes. An ionic liquid, 1-butyl-2,3-dimethylimidazolium hexafluorophosphate, was found to show a better performance than Nafion when used as a coating film to protect silver chloride on Ag wire and support the cell membrane.

With prolonged implantation, biological fouling of electrochemical probes occurs [141]. In long-term FSCV studies, biofouling appears as a shift in the peak oxidative potential of the background signal that deteriorates over days or weeks, reducing the neurotransmitter sensitivity and selectivity. The increase in electrochemical impedance that occurs during long-term brain implantation makes the two-electrode configuration unsuitable for long-term studies. To prevent this problem, a three-electrode configuration with a platinum wire counter-electrode was developed that compensated for the increased electrochemical impedance and reduced peak shift in vivo. The three-electrode configuration retained DA sensitivity at artificially elevated impedance levels in vitro. While the use of the three-electrode configuration reduces the biofouling impedance component, the cathodic polarization of the Ag/AgCl reference electrode remains a problem. It has been shown that the Nafion coating of the reference electrode delays the onset of polarization.

In recent years, the field of sensor modification using antifouling materials has expanded significantly, and a large number of approaches have appeared that allow modification of the electrode surface. However, the main problem to be solved is the decrease in the sensitivity of sensors after the modification of electrodes with antifouling materials due to their poor-conductivity antifouling polymers. Moreover, there is also low mechanical stability, which is very important in in vivo studies, when using some modification methods, such as redox reactions, for the deposited polymers.

## 4. Perspectives and Conclusions

In general, electrochemical sensors have a number of advantages: (1) they do not require long-term sample preparation; (2) the electrical signal at the characteristic potential is selective, so it is possible to detect several substances; (3) their ability to be used in analysis in various living systems and biological fluids, including sweat, serum, urine, and cell culture medium; and (4) their ability to conduct in vivo measurements inside tissues.

Based on the results of research over the last four years, the following trends can be observed in the development of electrodes. First is the use of antibiofouling coatings; alternatively, surface modifiers can be used to increase the catalytic activity, thereby removing the requirement for antibiofouling coatings. Second is the development of multimodal approaches that bring together information from different physiological phenomena. Third is in vivo measurement of freely moving mice/rats or those that are conscious, thereby facilitating studies that are closer to natural conditions.

Despite the rapid developments in this area, there are some limitations regarding the use of electrochemical methods in the analysis of neurotransmitters. First, it is necessary to take into account the electrical activity of the analyte of interest, namely, the signal and background drift caused by electrode contamination. Second, due to the lower spatial resolution and size of electrodes, only extracellular signals at moderate levels and of a certain cell type can be determined. To overcome these limitations, the most suitable electrode materials are selected, such as silicon, ceramics, and polymeric substrates. The modification of the electrode surface is also used to improve the adsorption efficiency, improve the sensitivity, improve the electron transfer efficiency, and eliminate interference. Signal drift often occurs during continuous acquisition. The development of on-site or online calibration technology guarantees more reliable monitoring results. Accurate measurements are achieved by modifying the microelectrode surface using highly selective recognition ligands. Efforts have been made to improve both the electrode modification strategies and the electrode modification layers to ensure assembly stability and the long-term stability of the electrodes. The potential-free detection method allows for simultaneous electrophysiological recordings. These methods are expected to be applied to the analysis of neurotransmitters in the future.

Similar problems are encountered in the detection of almost all analytes under in vivo conditions. The organism is a challenging environment for detecting various analytes because of their low concentrations. Therefore, when creating sensors, the main areas that need to be improved are the selectivity, sensitivity, and accuracy. It is also necessary to improve the manufacturing methods for the production of simple and miniaturized sensors. The ideal electrode should be small in size but, at the same time, have high sensitivity. It is important to minimize damage to the tissue of subjects (animals and patients) during the implantation process and not worsen the condition of the living object. The integration of sensors with signal transmission systems may be useful for interpreting the relationship between changes in the bioanalyte concentrations and the onset and development of diseases during long-term measurements.

To achieve a higher selectivity, sensing elements such as aptamers, enzymes, and ion-permeable membranes have been developed for bioanalyte detection. At the same time, modification using nanomaterials (metal nanoparticles, carbon nanotubes, graphene, etc.) can improve the sensitivity of sensors. Long-term monitoring has been made possible by implantable and wireless sensors. Long-term implantable sensors must be stable to various temperature and environmental factors and not cause tissue inflammation or discomfort to animals, thereby affecting the results of measurements.

Modern emerging materials are being used to achieve significant progress in the development of electrochemical in vivo microsensors. Increased electron transfer rates, increased hydrophilicity, and changes the nature of the surface porosity have been achieved, and in connection with this, the sensitivity, selectivity, and protection against fouling of the electrochemical microsensor have significantly increased.

It is expected that with the development of a new material or functionalization method, more bioanalytes can be effectively detected in vivo. In addition, new opportunities will emerge with the development of soft materials and degradable materials that will aid in the long-term tracking of bioanalytes. All of these microsensors can help us better understand physiological and pathological processes.

Wearable and implantable electrochemical sensors are commonly used for clinical applications. They are easily introduced into people’s lives, allow noninvasive determination of analytes, and receive feedback in real time. Using these sensors, it will be possible to track dynamic changes in physiological components. The rapid development of microelectronics contributes to the development of these sensors. However, the development of wearable and implantable sensors is at an early stage. For clinical use, long-term stability and biocompatibility issues need to be addressed. Therefore, in order to create reliable flexible electrochemical sensors with multifunctionality and excellent biocompatibility, more efforts should be made to synthesize nanomaterials with excellent mechanical properties and electrochemical activity for clinical practice. The future development of electrochemical sensors should focus on stability, versatility, and miniaturization to meet the various requirements of medical diagnostics.

## Figures and Tables

**Table 1:** Detection neurotransmitters in vivo.

	Sensor Structure	Method	Limit of Detection	Linear Concentration Range	Animal/Area	Ref.
**DA**	Nafion-modified glass-sealed Au nanoelectrode	Amperometry	5.2 nM	0.01–2.55 μM	(Rat) brain, striatum	[57]
**DA**	CF disk electrode in the middle of the ring-disk microelectrode modified by Prussian blue (PB) and poly(2,3-dihydrothieno-1,4-dioxin) (PEDOT)	Amperometry	0.18 μM	0.5–25 μM	(Rat) brain, nucleus accumbens	[65]
**DA**	PTA-doped nanoporous conductive membrane-coated CFE	Amperometry	-	5–30 μM	(Rat) brain, nucleus accumbens	[66]
**DA**	Carbon nanotube fiber Nafion-modified	Potentiometry	5 nM	5–185 nM	(Mouse) brain, striatum	[68]
**DA**	Alkyl chain functionalized CFE modified by aptamer cholesterol amphiphiles	Amperometry	0.5 μM	0.5–2 μM	(Rat) brain, striatum, NAc	[69]
**DA**	3D-printed CFE	FSCV	11 nM (spheric) 10 nM (conical)	0.01–10 μM	(Rat) brain, caudate putamen	[71]
**Serotonin**	CFE modified by Nafion	FSCV	-	-	(Mouse) brain, CA2 region, substantia nigra, pars reticulata	[75]
**Serotonin**	CFE modified by Nafion	FSCV	-	-	(Mouse) brain, CA2 region of the hippocampus	[76]
**Choline**	Nanopipette filled with 1,2-1,2-dichloroethane containing THATPB	Amperometry	0.37 μM	1–54 μM	(Rat) brain, frontoparietal cortex	[77]
**H_2_S**	Ag_2_S/AgNP/CFE	Potentiometry	0.8 μM	2.5–160 μM	(Rat) brain, hippocampus	[82]

**Table 2:** Detection of ROS/ RNS in vivo.

	Sensor Structure	Method	Limit of Detection	Linear Concentration Range	Animal/Area	Ref
**NO**	CF modified with NiTSPc and Nafion	DPV	0.34 µM	0.5–10 µM	Zebrafish embryos	[90]
**NO**	CF modified with DNA-G4/porphyrin	DNPV	13.5 pM	100 pM–5 µM	(Mouse) Tumor	[91]
**NO**	CF modified with nickel porphyrin and fluorinated xerogel	DNPV	12.1 ± 3.4 nM	-	(Rat) Brain	[92]
**NOO^−^**	CF modified with HEMF		12.1 ± 0.8 nM	20.0 nM–2.0 μM	(Rat) Brain	[94]
**H_2_O_2_**	CF modified with 1,3-phenylenediamine	FSCV	-	-	(Rat) Brain	[95]
**H_2_O_2_**	Pt nanopipette-based nanoelectrode	Amperometry	-	0.1–100 µM	(Mouse) Tumor	[40]
**H_2_O_2_**	CF disk electrode in the middle of the ring-disk microelectrode modified by PB and PEDOT	Amperometry	0.4 µM	1–29 µM	(Rat) Brain	[65]

## Abbreviations

AA	ascorbic acid
**a**CSF	artificial cerebrospinal fluid
AD	Alzheimer’s disease
ChOx	choline oxidase
CFE	carbon fiber electrode
CFME	carbon fiber microelectrode
CNS	central neural system
CV	cyclic voltammograms
DA	dopamine
DPV	differential pulse voltammetry
DNPV	differential normal pulse voltammetry
EOGO	electrochemically oxidized graphene oxide
FC	frontal cortex
FDCA	2,5-furandicarboxylic acid
FIB	focused ion beam
FSCAV	fast scan cyclic adsorption voltammetry
FSCV	fast scan cyclic voltammetry
GC	glassy carbon
LM	leukocyte membrane
LLIM	liquid/liquid interface microsensor
LOD	limit of detection
MFB	medial forebrain bundle
NAc	nucleus accumbens
NE	norepinephrine
PANI	polyaniline
PD	Parkinson’s disease
PEDOT	poly(2,3-dihydrothieno-1,4-dioxin
PTA	polytannic acid
SD	spreading depolarization
SECM	scanning electrochemical microscopy
SICM	scanning ion-conductance microscopy
SNM	silicon dioxide membrane
SWNTs	single-walled carbon nanotubes
UA	uric acid
